# Investigating the role of MicroRNA-519d-3p in enhancing chemosensitivity of colorectal cancer cells to 5-Fluorouracil through PFKFB3 targeting

**DOI:** 10.1016/j.clinsp.2025.100606

**Published:** 2025-02-26

**Authors:** Yangyang Zhang, Yiqing Zhang, Yanan Xiao, Shufen Xu, Jie Li, Juan Li, Lisha Chang, Jie Ding, Di Wu, Li Wang, Guangxu Xu, Keming Wang

**Affiliations:** a Department of General Practice, The First Affiliated Hospital with Nanjing Medical University, Nanjing City, Jiangsu Province, PR China; b Department of Oncology, The Second Affiliated Hospital of Nanjing Medical University, Nanjing City, Jiangsu Province, PR China; c Professor, Department of Rehabilitation Medicine Center, The First Affiliated Hospital with Nanjing Medical University, Nanjing City, Jiangsu Province, PR China; d Department of Rehabilitation Medicine Center, Yixing People's Hospital, Yixing City, Jiangsu Province, PR China

**Keywords:** MicroRNA-519d-3p, Colorectal Cancer, 5-Fluorouracil, Chemosensitivity, PFKFB3, Therapeutic Approach

## Abstract

**Objective:**

In the fight against Colorectal Cancer (CRC), chemotherapy resistance is a major obstacle. Therefore, it is imperative to identify effective biomarker therapeutics. Despite microRNAs (miRs) playing a crucial role in drug resistance, the mechanisms comprising miR-519d-3p's role in CRC drug resistance have not been fully understood. Therefore, the present study aimed to investigate the biological function of miR-519d-3p in the chemosensitivity of CRC cells to 5-Fluorouracil (5-FU).

**Methods:**

CRC cells were treated with 5-FU and transfected. Cellular proliferation, invasion, and apoptosis were evaluated. The relationship between miR-519d-3p and 6-Phosphofructokinase-2/Frucose-2, 6-Biphosphatase-3 (PFKFB3) was analyzed, and their interaction in CRC was further investigated. In vivo tumor experiments were conducted to investigate the function of miR-519d-3p and 5-FU in CRC.

**Results:**

As determined, CRC cells overexpressing miR-519d-3p were more sensitive to 5-FU in vitro, as miR-519d-3p inhibits proliferation and invasion and stimulates apoptosis. miR-519d-3p directly targeted PFKFB3. In CRC cells, PFKFB3 overexpression rescued miR-519d-3p-induced 5-FU toxicity. In vivo results showed that mice co-treated with miR-519d-3p mimics and 5-FU showed higher antitumor activity.

**Conclusion:**

Overall, it may be possible to improve 5-FU chemosensitivity of CRC cells by targeting miR-519d-3p and PFKFB3.

## Highlights

• miR-519d-3p affects the antitumor effect of 5-FU on CRC cells.
• miR-519d-3p could regulate PFKFB3 and could have a significant effect on CRC.
• PFKFB3 attenuated 5-FU sensitivity in CRC cells and enhanced glycolysis.

## Introduction

Globally, the third most common kind of cancer worldwide is Colorectal Cancer (CRC) which has been treated with 5-Fluorouracil (5-FU) as a first treatment option for over 60 years.^1^^,^^2^ 5-FU, however, has been shown to be ineffective mainly because of acquired drug resistance, while the mechanisms underlying 5-FU resistance acquisition are complex and are commonly mediated by complicated processes.^3^^,^^4^ It is therefore necessary to study the mechanisms underlying resistance to 5-FU drugs.

Additionally, it was previously reported that the dysregulation of microRNAs (miRNAs/miRs) is involved in cancer progression, as well as cancer cells' resistance to therapies.^5^^,^^6^ In this paper, the authors initially conducted correlation sequencing on 5-FU-resistant and non-resistant CRC cell strains. The outcomes indicated that miR-519d-3p was markedly different. In the past few years, numerous studies have verified that miR-519d-3p affects the development of a diverse array of malignant tumors. Liang et al. showed that miR-519d has the potential to regulate the Wnt/β-catenin signaling pathway in pancreatic malignancies, thereby inhibiting tumor growth.^7^ Li et al. found that miR-519d-3p can inhibit gastric cancer by down-regulating the expression of B-cell lymphoma6 cell proliferation and invasion.^8^ Relevant studies further found that miR-519d-3p has an important impact on improving tumor chemotherapy resistance. Jiang et al. pointed out that miR-519d-3p improves the sensitivity of cervical cancer cells to cisplatin by inhibiting the PI3K/AKT signaling pathway.^9^ Chen et al. showed that miR-519d-3p regulates CREB1 expression and mediates CRC chemosensitivity to oxaliplatin.^10^ Therefore miR-519d-3p may inhibit tumor growth and alleviate the disease by improving tumor chemotherapy resistance, but the effect and mechanism of action in regard to the sensitivity of miR-519d-3p of CRC to chemotherapy with 5-FU is still unclear and needs further study.

Based on database bioinformatics (http://starbase.sysu.edu.cn/), we predicted that miR-519d-3p might be capable of focusing and binding multiple downstream target genes, among which PFKFB3 showed strong binding affinity. In comparison to the other PFKFB isozymes, the protease that is encoded by the PFKFB3 gene has the greatest ratio, and it has the ability to efficiently increase the efficiency of glycolysis in cells.^11^ So does miR-519d-3p have any effect on glycolysis? For this purpose, the authors reviewed the relevant literature: Sun et al. proposed that PVT1 could function as an oncogenic lncRNA, thereby promoting tumor progression and glycolysis by competing with miR-519d-3p to regulate HIF-1A^12^; Another report demonstrated that the H19/miR-519d-3p/LDHA axis predominantly contributed to aerobic glycolysis, proliferation, and immune evasion of GC cells.^13^ In recent years, the expression of PFKFB3 being elevated in cancerous tissues and the role that it plays in the process of carcinogenesis have attracted attention while several studies have confirmed that PFKFB3 is overexpressed in breast cancer, CRC, pancreatic cancer, gastric cancer are overexpressed, leading to tumor cell proliferation, distant metastasis, and reduced patient survival.^14^^,^^15^ In addition, PFKFB3 was revealed to play an essential function in tumor chemosensitivity, Long et al. demonstrated The triggering of the PFKFB3/HIF-1α axis promotes drug resistance in hepatocellular carcinoma cells.^16^ According to the overexpression, Deng et al. found of PFKFB3 decreased chemosensitivity to 5-FU in LoVo, HCT116 cells.^17^ Therefore the relationship among miR-519d-3p with PFKFB3, and the mechanism of action of both in the sensitivity of CRC to 5-FU chemotherapy needs to be explored in more depth.

In summary, the authors hypothesized the following ideas: 1) miR-519d-3p may inhibit tumor growth by improving tumor chemotherapy resistance, but there is a lack of relevant studies on the sensitivity of miR-519d-3p to 5-FU in CRC cells; 2) Based on the prediction of bioinformatics databases, PFKFB3 may be a target gene of miR-519d-3p, and PFKFB3 has been similarly confirmed to be closely related to tumor growth and chemotherapy sensitivity, but there is a lack of relevant studies on the mechanism of their functions in the sensitivity of CRC to 5-FU chemotherapy, as well as the relationship between miR-519d-3p and PFKFB3. Therefore, this paper was developed at the following levels: 1) We constructed 5-FU resistant cell lines, performed gene sequencing with non-resistant cell lines and RT-PCR to verify the genetic differences of miR-519d-3p; 2) We verified the effect of miR-519d-3p on the sensitivity of CRC cells to the action of 5-FU; 3) In vitro experiments, we identified the consequences of miR-519d-3p on the regulation of PFKFB3; 4) In vitro and in vivo experiments the authors observed the effect of miR-519d-3p targeted regulation of PFKFB3 on the sensitivity of CRC 5-FU action, in order to establish novel targets and directions for the diagnostic and therapeutic mechanisms of CRC.

## Materials and methods

### Cell culture

The human CRC cell lines HT-29 and DLD-1 were acquired from FU Heng Biology (Shanghai, China). The normal human colon mucosal epithelial NCM460 cell line was established by Yuchun Co., Ltd. (Shanghai, China), and HCT116 cells were generously provided by Dr. Tan (Department of Traditional Chinese Medicine, Jiangsu, China). The cell lines mentioned above were cultured in RPMI-1640 medium that was supplemented with 10 % fetal bovine serum (FBS; Gibco; Thermo Fisher Scientific, Inc.), 1 % penicillin (10,000 U/mL), and streptomycin (10 mg/mL). These cells were maintained in a humidified atmosphere with 5 % CO_2_ at 37 °C.^18^

HT29 and DLD-1 human colon cancer cell lines were obtained from Wuhan Pricella Life Science and Technology Co., Ltd. in order to generate 5-FU resistant cell lines. The cells were dispersed into six-well plates. The cells were treated with 5-FU at concentrations of 0, 12.5, 25, 50, 100, 200, and 300 μg/mL for 1 h, respectively, when the cell density reached 70 %‒80 %. The cells were rinsed twice with PBS following drug treatment. Fresh medium was substituted for the drug-containing medium, which was devoid of 5-FU. At the second transit of the cells, the drug-containing medium was reintroduced, and the handling time was progressively extended to 2, 3, 6, 12, 24, 36, 48, 60, and 72 h. The subsequent action was identical to the one that was previously described. To verify the successful generation of 5-FU-resistant cells, a CCK-8 assay was conducted until the sensitivity of cells to 5-FU treatment was markedly reduced and stabilized.^19^^,^^20^

### RNA isolation and sequencing

The total RNA concentration was determined using a Nanodrop (ND-1000) and RNA was isolated from Cell using TriReagent (Ambion Inc, TX) in accordance with the manufacturer's protocol. The quality was evaluated using the Agilent 2100 Bioanalyzer, and samples with an RIN value of 7 or higher were selected for further analysis. The Illumina® TruSeq™ Small RNA Sample Preparation protocol was employed to generate small RNA sequencing libraries. Sequencing Data Analysis and Normalization were conducted in accordance with the methodology previously described.

### Cell transfection

The miR-519d-3p mimic and inhibitor were acquired from GenePharma (Suzhou, China). Lipofectamine 2000 (Invitrogen, Carlsbad, California) was employed to conduct transfection in accordance with the manufacturer's instructions.miR-519d-3p mimic sequence: 5′CAAAGUGCCUCCCUUUAGAGUG3’, miR-519d-3p inhibitor sequence: 5′CACUCUAAAGGGCAUUUG3’,miR-519d-3p negative control sequence: 5′UCUACUUUCUAGGAGGUUGUGA3’.

Small interfering RNA targeting PFKFB3(si-PFKFB3): PFKFB3 (human)-siRNA-1–1529: 5′GGAUAAGAGUGCAGAGGAGTT3’, pcDN-A-PFKFB3 overexpression plasmid (pCMV-PFKFB3(human)-EGFP-Neo.dna), negative control plasmids (pCMV-EGFP-Neo.dna (vector) sequence: 5′UUCUCCGA-ACGUGUCACGUTTACGUGACACGUUCGAGAATT3’ were purchased from General Biology Co., Ltd. Prior transfection, the quality/concentration of nucleic acid transfection by siPFKFB3 was 1.28 μg/mL, the quality/concentration of pcDNA-PFKFB3 transfection nucleic acid was 4 μg/mL, the nucleic acid quality/concentration of miR-519d-3p mimic and inhibitor was 1.28 μg/mL. A total of 10^5^ DLD-1 and HT-29 cells in a complete culture medium were seeded into 24-well plates and cultured until they reached 70 %‒80 % confluence. Cell transfection was then carried out using Lipofectamine® 3000 (Invitrogen; Thermo Fisher Scientific). At 24 h or 48 h following transfection, follow-up experiments were performed.

### Cell viability assay

CRC cells were distributed into 96-well plates at a density of 4 × 10^3^ cells per well and cultured for 24 h. HT-29 and DLD-1 cells were subsequently treated with 5-FU at concentrations of 50, 100, 200, 400 and 800 µg/mL (Meilunbio Co., Ltd.). The absorbance was measured at 450 nm after the treatment was administered for 24 h. The corresponding half-maximal Inhibitory Concentration (IC_50_) was calculated using GraphPad Prism software, and the 5-FU inhibition rate (%) was determined. The cell viability was evaluated using a Cell Counting Kit-8 assay (Wuhan Servicebio Technology Co., Ltd).^21^^,^^22^

### Colony formation assay

1 × 10^3^ HT-29 and DLD-1 cells were digested with pancreatic enzymes. Following exposure to 5-FU (50 μg/mL), The cells were cultured in 6-well plate, the fresh medium was added every 3-days. After 14-days, the cells were fixed with 4 % paraformaldehyde for 30 min. Subsequently, the colonies were stained with crystal violet dye, counted, and observed under a microscope.^23^

### Transwell invasion assays

A chamber with 8.0-μm pore size membranes was used to transfer a total of 2 × 10^5^ cells. The upper chamber was coated with 40 μL Matrigel attenuated in medium, while the lower chamber was supplemented with DMEM with 20 % FBS (Gibco; Thermo Fisher Scientific, Inc.). After incubation at 37 °C for 24 h, the upper surface of the membrane was fixed with 20 % methanol for 20 mins, and crystal violet was used to stain the cells. The inverted light microscope was used to acquire cell images at a magnification of × 200.^24^

### Cell apoptosis assay

After 24 h of transfection, 5-FU (50 μg/mL) was administered to CRC cells, and the apoptosis rate was evaluated using the Fluorescein Isothiocyanate (FITC) Annexin V Apoptosis Detection Kit (BD Biosciences). 500 μL of 1 × binding buffer was used to re-suspend the cells, and the cells were incubated with 5 μL of Annexin V-FITC and 5 μL of Propyl Iodide (PI) solutions at ambient temperature for 15 mins. Lastly, the FACScan flow cytometer (BD Biosciences) was employed to quantify the percentage of apoptotic cells. The apoptosis is the sum of the early and late stages. The sum of early and late apoptosis rates is depicted in the bar chart.^25^

### Reverse transcription-quantitative pcr (RT-qPCR)

A TRIzol reagent mix (Wuhan Servicebio Technology Co., Ltd.) was employed to isolate total cellular RNA from CRC cells. RT-qPCR was used to reverse transcribe the total RNA into cDNA. The following thermocycling conditions were employed for qPCR: 40 cycles of 95 °C for 15 ss and 60 °C for 30 ss, followed by a 30 second hold at 95 °C. U6 and β-actin were employed as internal controls for miRNA and mRNA, respectively. The 2^−ΔΔCt^ method was employed to quantify the relative RNA expression levels.^26^ Table 1 contains the primer sequences that were employed.

**Table 1:** Primer sequences for reverse transcription-quantitative PCR.

Gene	5′‒3′	3′‒5′
miR-519d-3p	ACACTCCAGCTGGGCAAAGTGCCTCCCTTT	AATCTCACGAGTTGACTTAACGGCTGAGGTGCTGTGGTCAACTC
PFKFB3	TGCCTGCTTGCCTACTTCCTG	TTTCTTCCCTGGATTGGGCGA
U6	CTCGCTTCGGCAGCACA	TGCGTTTAAGCACTTCGCAA
β-actin	CACCCAGCACAATGAAGATCAAGAT	CGAACTGAGTCCTAAATTTTTGACC

### Western blot analysis

An extraction reagent (Nanjing KeyGen Biotech Co., Ltd.) was employed to extract total cellular proteins. Subsequently, approximately 30 μg of protein extracts were separated using SDS-PAGE and subsequently transferred to PVDF membranes. Following this, the membranes were initially incubated with primary antibodies against PFKFB3 (1:1000; 13,763–1-AP, ProteinTech) and β-actin (1:1000; GB15003, Wuhan Servicebio) at 4 °C overnight. The membranes were subsequently washed with 0.1 % TBS-Tween-20 three times. The membranes were subsequently incubated with the corresponding secondary antibody, Goat Anti-Rabbit IgG (1:1,0000; 111–035–003, Jackson Immuno Research Laboratories, Inc.), diluted in PBS, for 2 h. Chemiluminescence was employed to observe the protein bands.

### Lactate production assay

The cells were cultured in 6-well plates for 24 h. The lactate content in cells was determined using the corresponding lactate reagent (General Biologicals Corporation), ,^27^ and the supernatant was extracted the following day.

### Luciferase reporter assay

For 24 h, CRC cells were co-transfected with the PFKFB3-Wild Type (WT) or PFKFB3-Mutant (MUT) luciferase reporter plasmids and miR-519d-3p mimics or control mimics. A luciferase activity assay reagent (General Biologicals Corporation) was employed to measure the luciferase activity.

## Tumor xenograft model

For the establishment of a tumor xenograft mouse model, 4-week-old male BALB/c nude mice were injected with 5 × 10^6^ DLD-1 cells in 0.1 mL PBS subcutaneously in the flank. The experiment ended without anyone dying. All nude mice in the experiment were euthanized with CO_2_. The mice were monitored daily for condition, diet, and behavior. Tumor size was measured every two days, starting from day-7 following subcutaneous tumor implantation. Furthermore, mice were intraperitoneally injected with 20 mg/kg 5-FU (every 2-days) and 25 mg/kg PFK15 (every 3-days). Prior to injection, cells were transfected with 10*g* miR-519d-3p mimics. Following 14 days, mice were euthanized by 100 % carbon dioxide at a flow rate of 30 % volume displacement per minute, according to the 2020 American Veterinary Medical Association guidelines. Tumor weight should not exceed 10 % of body weight, while the average tumor diameter should not exceed 15 mm. Tumor tissues were extracted promptly, measured and images were captured under a microscope. Volume (V) = tumor length (L) × tumor short length (W2)/2. Histological analysis of paraffin-embedded tissues was performed with Hematoxylin and Eosin (H&E). Research conducted on animals was approved by Nanjing Medical University's Animal Experiment Center (approval n° 2,203,044). This study follows ARRIVE guidelines.

## EdU incorporation assay

An EdU kit was purchased from Meilunbio. In summary, cells were dispersed into 96-well plates and cultured overnight. Following the corresponding treatment, the cell suspension was supplemented with 1 × EdU solution for 2 h followed by cell fixation for 15 min. After washing, the reaction solution was configured and incubated for 30 min. Following washing, the EdU positive cells (red) were observed under a fluorescence microscope.^28^

### Gene expression profiling interactive analysis (GEPIA)

GEPIA (http://gepia.cancer-pku.cn/) database was employed to analyze the differential expression of PFKFB3 between CRC tissue and normal tissue, and the correction between the differential expression of PFKFB3 and the prognosis of CRC patient prognosis.

### MiRNAs data analysis

MiRNAs analysis was performed by Next Generation Sequencing. Differentially Expressed (DE) miRNAs in the sequences were screened using the *R* package limma; *p* < 0.05 were considered the cutoffs for DE miRNAs.^29^^,^^30^

### Statistical analysis

Graphpad Prism 8 (GraphPad Software, Inc.) and SPSS version 24.0 (IBM Corp.) have been used to conduct all statistical analyses. Continuous variable data in a normal distribution were compared using the *t*-test (two groups) or the ANOVA test (three or more groups). Measurements are presented as mean Standard deviation (S). The data were analyzed using Tukey's multiple comparison test for multi-group comparisons. The threshold for statistical significance was set at *p* < 0.05.

## Results

### Construction of 5-FU resistance model and miR-519d-3p differential expression analysis

**Fig. 1:**
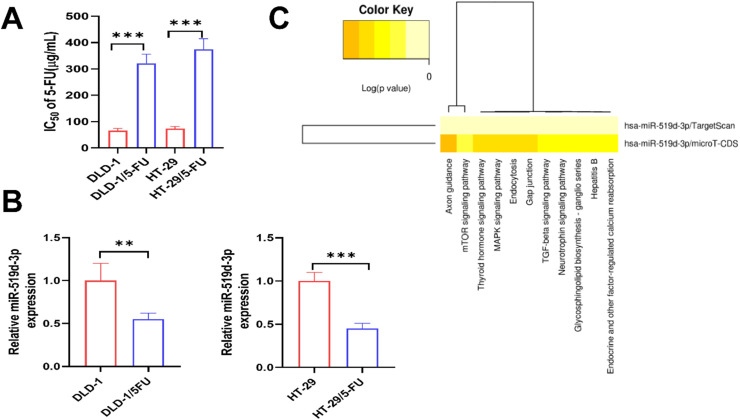
**Construction of 5-FU resistance model and miR-519d-3p differential expression analysis.** (A) CCK8 essay was performed to examine the IC_50_ value for DLD-1, HT-29, DLD-1/5FU and HT-29/5-Fu cells after 5-fu treatment. (B) The expression levels of miR-519d-3p in the HT-29, and HT-29/5-Fu cells by RT-qPCR. (C) Statistically significant correlations were revealed between miR-519d-3p and their mediated pathways by p-value (log scaled) in a heatmap. Red represents high significance. * *p* < 0.05, ** *p* < 0.01, *** *p* < 0.001.

In order to investigate the variations in miR-519d-3p levels through sequencing, the authors initially established 5-FU-resistant strains and non-resistant strains as the 5-FU RS group and NRS group. The results of CCK-8 demonstrated that the IC_50_ of DLD-1 and HT-29 non-resistant strains was 63.18±8.11 μg/mL and 73.44±8.55 μg/mL, respectively. In contrast, the IC_50_ of DLD-1 and HT-29 resistant strains was 320.90±35.12 μg/mL and 374.35±40.32 μg/mL, respectively. These results suggest that 5-FU drug-resistant cells were successfully constructed, and the resistance of DLD-1 and HT-29 resistant strains was 5.079 and 5.093 times that of non-resistant strains, respectively (Fig. 1A). The sequencing analysis table indicated that there were substantial distinctions between miR-519d-3p drug-resistant strains and non-drug-resistant strains (Table 2). In contrast, the content of miR-519d-3p in drug-resistant strains was lower than that in non-drug-resistant strains as detected by RT-qPCR (Fig. 1B). A series of bioinformatics databases were analyzed, and it was discovered that miR-519d-3p were implicated in numerous biological pathways (Fig. 1C). miR-519d-3p regulated numerous target genes in this pathway (Table 3). These data indicated the significant functions of mir-519d-3p in CRC and CRC chemo-resistance.

**Table 2:** Data of the microarray between HT-29 and HT-29/5-FU tumor cells.

A, Up-regulated miRs		
MiR name	p-value	Fold change
hsa-miR-1269a	0.00000	7.83231
hsa-miR-1269b	0.00000	5.75393
hsa-miR-181a-2–3p	0.00000	3.21837
hsa-miR-30e-3p	0.00000	2.1588
hsa-miR-30a-3p	0.00000	2.12918
hsa-miR-548az-5p	0.00000	4.29139
hsa-miR-629–5p	0.00000	1.45936
hsa-miR-30c-2–3p	0.00000	3.16602
hsa-miR-22–3p	0.00000	1.58723
hsa-miR-99b-3p	0.00000	1.26611
hsa-miR-1262	0.00000	2.17112
hsa-miR-182–5p	0.00000	1.15835
hsa-miR-1286	0.00000	1.88006
hsa-miR-21–3p	0.00000	1.19472
hsa-miR-183–5p	0.00000	1.11234
hsa-miR-28–3p	0.00000	1.11292
hsa-miR-3187–3p	0.00000	3.01561
hsa-miR-1275	0.00000	1.7711
hsa-miR-151a-3p	0.00000	1.06485
hsa-miR-10,395–3p	0.00000	1.16417
hsa-miR-99b-5p	0.00000	1.0291
hsa-miR-30b-5p	0.00000	1.03523
hsa-miR-30c-1–3p	0.00000	1.97104
hsa-miR-584–5p	0.00000	1.04564
hsa-miR-378c	0.00000	1.04705
hsa-miR-185–3p	0.00000	1.10073
hsa-miR-203b-3p	0.00001	1.00832
hsa-miR-143–3p	0.00002	1.89288
hsa-miR-4791	0.00003	1.32104
hsa-miR-548bc	0.00008	1.35452
hsa-miR-183–3p	0.00008	2.41157
hsa-miR-106b-3p	0.00009	0.80889
hsa-miR-151a-5p	0.00013	0.85355
hsa-miR-135b-3p	0.00015	1.45467
hsa-miR-552–3p	0.00015	0.82327
hsa-miR-215–5p	0.00016	0.78515
hsa-miR-3605–3p	0.00035	6.68044
hsa-miR-378d	0.00037	1.19805
hsa-miR-941	0.00102	0.69559
hsa-miR-1266–5p	0.00107	0.86453
hsa-miR-181b-5p	0.0012	0.70647
hsa-miR-548aq-3p	0.00125	2.24974
hsa-miR-4660	0.00127	6.4898
hsa-miR-579–5p	0.00127	6.4898
hsa-miR-330–3p	0.00143	0.96721
hsa-miR-320c	0.00149	2.76064
hsa-miR-4664–3p	0.00171	0.79659
hsa-miR-3128	0.00187	1.18254
hsa-miR-320b	0.00192	1.62844
hsa-miR-3177–3p	0.00217	1.63646
hsa-let-7e-5p	0.00305	0.61223
hsa-miR-1–3p	0.00361	1.69638
hsa-miR-1307–5p	0.00488	0.79573
hsa-miR-573	0.00548	1.18665
hsa-miR-30e-5p	0.0056	0.59253
hsa-miR-197–3p	0.00638	0.59837
hsa-miR-629–3p	0.00779	1.71855
hsa-miR-151b	0.00961	3.11799
hsa-miR-1843	0.01371	0.79412
Novel_21_1,079,249	0.01393	1.40986
hsa-miR-141–3p	0.01511	0.55952
hsa-miR-181a-5p	0.01618	0.51732
hsa-miR-584–3p	0.0173	5.86127
hsa-miR-4652–5p	0.01971	1.19148
hsa-miR-652–3p	0.02018	0.59675
hsa-miR-3679–5p	0.02222	1.25932
hsa-miR-140–3p	0.02303	0.47177
hsa-miR-532–5p	0.02387	0.47317
hsa-miR-149–5p	0.02854	0.65427
hsa-miR-4802–3p	0.02943	1.18746
hsa-miR-769–5p	0.02995	0.45191
hsa-miR-26a-5p	0.03323	0.43931
hsa-miR-145–5p	0.03362	5.50577
hsa-miR-3679–3p	0.03362	5.50577
hsa-miR-616–3p	0.03362	5.50577
hsa-miR-192–3p	0.03415	0.78324
hsa-miR-219b-5p	0.0347	1.93593
hsa-miR-338–3p	0.03848	1.04927
hsa-miR-3691–5p	0.03848	1.04927
hsa-miR-22–5p	0.04198	2.0882
hsa-miR-6505–5p	0.04198	2.0882
hsa-miR-3944–3p	0.04254	1.43987
hsa-miR-3145–3p	0.04431	2.82167
hsa-miR-320d	0.04431	2.82167
hsa-miR-653–5p	0.04431	2.82167
hsa-miR-4758–3p	0.04528	1.60157
hsa-miR-146b-5p	0.04556	0.51255
hsa-miR-2110	0.04617	0.68665
hsa-miR-192–5p	0.04656	0.40738
hsa-miR-27a-5p	0.04795	0.495
**B, Down-regulated miRs**		
**MiR name**	**p-value**	**Fold change**
hsa-miR-7974	0.00000	−2.33261
hsa-miR-519d-3p	0.00000	−3.4503
hsa-miR-1248	0.00000	−3.06218
hsa-miR-4521	0.00000	−1.37589
hsa-let-7*g*-5p	0.00000	−1.10095
hsa-let-7i-5p	0.00000	−1.05525
hsa-miR-92a-3p	0.00000	−1.0563
hsa-miR-20a-5p	0.00000	−1.03944
hsa-miR-200b-3p	0.00001	−0.91032
hsa-miR-191–5p	0.00002	−0.89438
hsa-miR-1303	0.00002	−1.20937
hsa-miR-374b-3p	0.00003	−1.61678
hsa-miR-1307–3p	0.00008	−0.84483
hsa-miR-1287–5p	0.00041	−1.3789
hsa-miR-577	0.00049	−1.24214
hsa-miR-335–5p	0.00069	−1.04265
hsa-miR-622	0.0007	−2.62858
hsa-miR-7–5p	0.0007	−0.69613
hsa-miR-103a-3p	0.00125	−0.66489
hsa-miR-492	0.00127	−3.74218
hsa-miR-1255a	0.00131	−1.33212
hsa-miR-141–5p	0.00149	−1.25628
hsa-miR-30d-3p	0.00197	−1.00783
hsa-miR-874–3p	0.00197	−1.22053
hsa-miR-421	0.00261	−0.79412
hsa-miR-17–5p	0.0037	−0.60894
hsa-miR-552–5p	0.00392	−0.71208
hsa-miR-576–3p	0.0043	−0.83863
hsa-miR-642a-5p	0.00444	−2.99438
hsa-miR-224–5p	0.00526	−0.5782
hsa-miR-671–5p	0.00532	−0.79301
hsa-miR-107	0.00543	−0.63028
hsa-miR-660–5p	0.00549	−0.87963
hsa-let-7f-5p	0.00769	−0.54718
hsa-miR-196a-5p	0.00901	−0.58218
hsa-miR-4454	0.01079	−0.8646
hsa-miR-196b-5p	0.01102	−0.55476
hsa-miR-425–5p	0.01176	−0.54634
hsa-miR-574–3p	0.01356	−0.59245
hsa-miR-130b-5p	0.01445	−0.80836
hsa-miR-580–3p	0.01608	−2.44497
hsa-miR-148b-5p	0.01611	−0.6425
hsa-miR-3654	0.01619	−1.85687
hsa-miR-335–3p	0.01715	−0.49545
hsa-miR-3613–5p	0.01813	−1.07413
hsa-miR-18a-3p	0.01879	−0.89444
hsa-miR-455–5p	0.02036	−0.52509
hsa-miR-4485–3p	0.02114	−1.48935
hsa-miR-93–5p	0.02184	−0.47471
hsa-miR-185–5p	0.02313	−0.47694
hsa-miR-32–3p	0.02504	−1.74224
hsa-miR-212–3p	0.02678	−2.29443
hsa-miR-1296–5p	0.02735	−0.72607
hsa-miR-339–5p	0.02921	−0.58047
hsa-miR-19b-3p	0.03191	−0.58198
hsa-miR-30d-5p	0.0322	−0.43898
hsa-miR-365a-3p	0.03249	−0.88748
hsa-miR-365b-3p	0.03249	−0.88748
hsa-miR-548n	0.03356	−0.90692
hsa-miR-10,394–5p	0.03362	−5.78323
hsa-miR-3916	0.03362	−5.78323
hsa-miR-4741	0.03362	−5.78323
hsa-miR-33a-3p	0.03484	−1.3839
hsa-miR-454–3p	0.03897	−0.47224
hsa-miR-500a-3p	0.04175	−0.54283
hsa-miR-152–3p	0.04845	−0.45099

**Table 3:** Statistical significance in pathways mediated by miR-519d-3p.

KEGG pathway	Genes	p-value
Axon guidance	19	0.00236
Thyroid hormone signaling pathway	13	0.00358
Endocytosis	25	0.00423
Gap junction	10	0.00519
MAPK signaling pathway	29	0.01339
Glycosphingolipid biosynthesis - ganglio series	3	0.01909
Hepatitis B	14	0.01926
Endocrine and other factor-regulated calcium reabsorption	7	0.02229
mTOR signaling pathway	9	0.02351
TGF-beta signaling pathway	10	0.02490
Neurotrophin signaling pathway	17	0.09170
Estrogen signaling pathway	11	0.09568
Renal cell carcinoma	9	0.11081
Arachidonic acid metabolism	1	0.11865
Glioma	8	0.16769
Circadian rhythm	6	0.17347
Bladder cancer	7	0.18883
Retrograde endocannabinoid signaling	10	0.18883
Prolactin signaling pathway	9	0.19286
Dopaminergic synapse	15	0.00005

### The impact of miR-519d-3p on the 5-FU chemotherapeutic response of crc cells

**Fig. 2:**
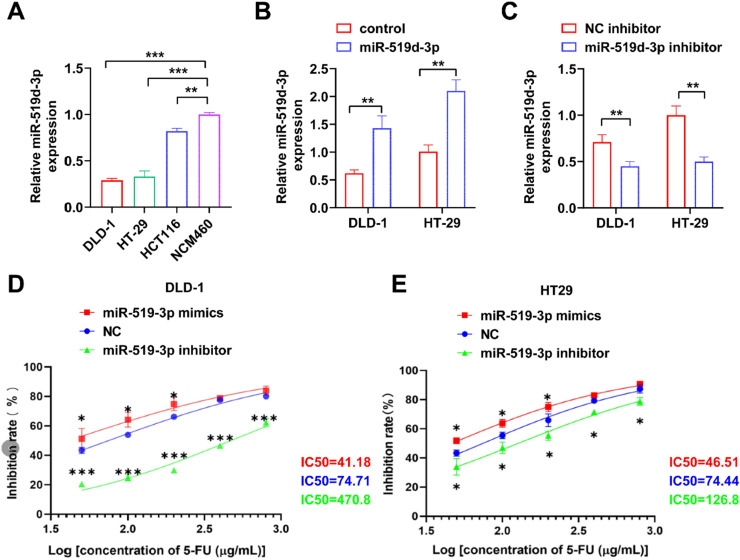
**miR-519d-3p regulates the chemosensitivity of CRC cells to 5-FU.** (A) The expression levels of miR-519d-3p in the CRC cell lines HT-29, DLD-1 and HCT116, and normal human colon mucosal epithelial NCM460 cell line were detected by RT-qPCR. (B and C) The expression levels of miR-519d-3p in DLD-1 and HT-29 cells transfected with miR-519d-3p mimics or inhibitor were detected using RT-qPCR. (D and E) CRC cells transfected with miR-519d-3p mimics or inhibitor were treated with different 5-FU doses. The inhibition rate of CRC cells was measured using a Cell Counting Kit 8 assay. * *p* < 0.05, ** *p* < 0.01, *** *p* < 0.001. Experiments were repeated at least three times. miR-519d-3p, microRNA-519d-3p; CRC, Colorectal Cancer; 5-FU, 5-fluorouracil; RT-qPCR, Reverse Transcription-Quantitative PCR.

Compared with NCM460, miR-519d-3p was expressed at lower levels in the CRC cell lines DLD-1, HT-29 and HCT116 (Fig. 2A). To examine the regulatory effect of miR-519d-3p overexpression on CRC, cells were transfected with miR-519d-3p mimics. qPCR validated the elevation of miR-519d-3p level (Fig. 2B). However, miR-519d-3p was downregulated after miR-519d-3p inhibitor transfection (Fig. 2C). For CRC cells overexpressing miR-519d-3p, chemosensitivity was enhanced (Fig. 2D). CRC cell lines were significantly less sensitive to 5-FU chemotherapy after silencing of miR-519d-3p (Fig. 2D and E).

### Impact of miR-519d-3p on the antitumor effect of 5-FU on crc cells

**Fig. 3:**
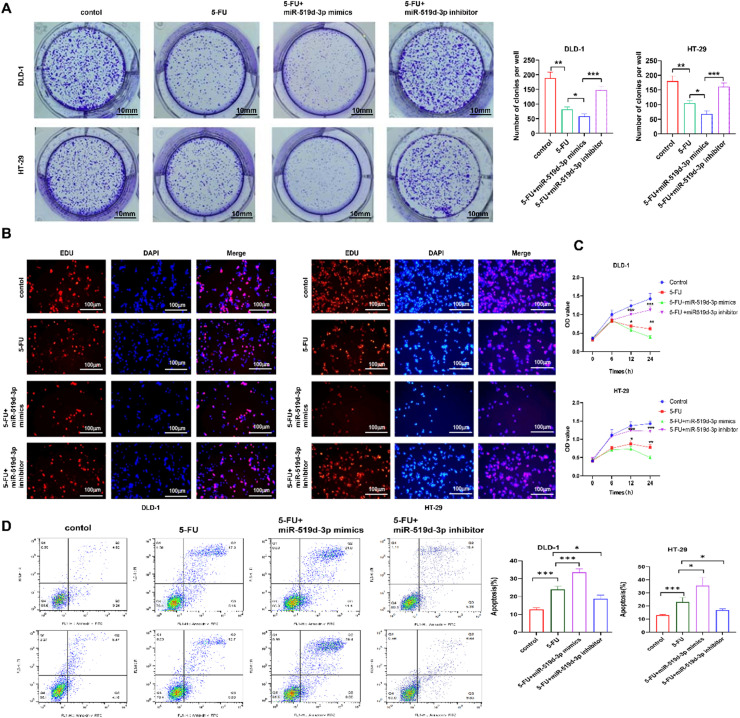
**miR-519d-3p enhances the antitumor activity of 5-FU against CRC cell proliferation, invasion and apoptosis.** (A) The proliferation ability of miR-519d-3p-overexpressing or -silencing 5-FU-treated CRC cells was assessed by colony formation and (B) EdU assays (magnification, × 200). (C) Colorectal cancer cells transfected with miR-519d-3p mimics or inhibitors were treated with different doses of 5-FU. The proliferation of CRC cells was measured using a cell counting kit 8. (D) Flow cytometric analysis was carried out to determine the proportion of apoptotic miR-519d-3p-overexpressing CRC cells treated with 5-FU. * *p* < 0.05, ** *p* < 0.01, *** *p* < 0.001. miR-519d-3p, microRNA-519d-3p; 5-FU, 5-Fluorouracil; CRC, Colorectal Cancer.

The effect of miR-519d-3p on 5-FU tolerance in CRC cells was further investigated by inducing or knocking down miR-519d-3p in HT-29 and DLD-1 cells. Further investigation of miR-519d-3p's effect on CRC cells' resistance to 5-FU was conducted by inducing or knocking down miR-519d-3p in HT-29 and DLD-1 cells. Therefore, following cell treatment with 5-FU, cell proliferation was reduced in miR-519d-3p-overexpressing CRC cells. However, cell transfection with miR-519d-3p inhibitor exhibited the opposite effect. Furthermore, the antitumor response of CRC cells to 5-FU was verified by colony formation, CCK-8 assay and EdU assays (Fig. 3A‒C). Flow cytometry assay confirmed that miR-519d-3p could induce cancer cell apoptosis to 5-FU, compared to the control (12.82 % and 11.62 % in CRC cells, respectively), and the cell apoptosis rate in the 5-FU group was 23.95 % and 23.05 %. More significantly, CRC cell apoptosis was notably enhanced in the miR-519–3p + 5-FU group (33.57 % and 42.20 % in CRC cells, respectively but the CRC cell apoptosis was repressed in the miR-519–3p inhibitor + 5-FU group (18.70 % and 16.77 % in CRC cells, respectively) as shown in (Fig. 3D). Overall, the foregoing data suggested that miR-519d-3p affects the antitumor effect of 5-FU on CRC cells.

### Regulatory role of miR-519d-3p in suppressing PFKFB3 expression

**Fig. 4:**
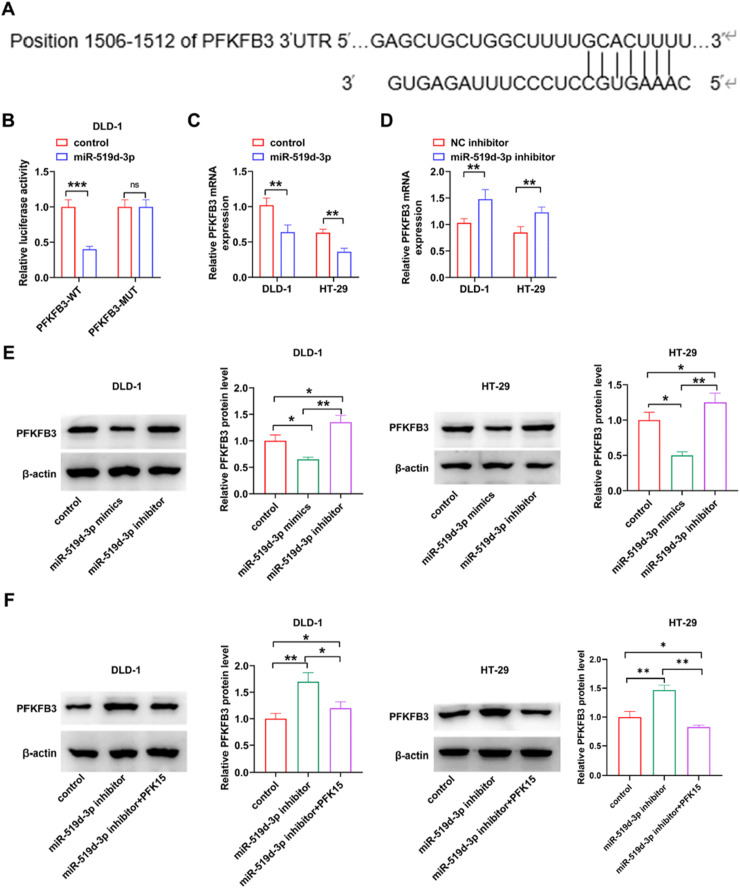
**miR-519d-3p acts as a tumor suppressor to regulate PFKFB3 in human CRC cells.** (A) TargetScan was used to predict the binding site of miR-519d-3p on PFKFB3 3′-UTR. (B) The luciferase activity was measured in DLD-1 cells co-transfected with miR-519–3p mimics and WT or mutant PFKFB3 3′-UTR. (C and D) Reverse transcription-quantitative PCR and (E) western blot analysis were performed to evaluate the effect of miR-519–3p overexpression or knockdown on the expression of PFKFB3. (F) The protein expression levels of PFKFB3 were determined in miR-519–3p-depleted PFK15-treated CRC cells using western blot analysis. * *p* < 0.05, ** *p* < 0.01, *** *p* < 0.001. miR-519d-3p, microRNA-519d-3p; PFKFB3, 6-Phosphofructokinase-2/Frucose-2, 6-Biphosphatase-3; WT, Wild-Type; 3′-UTR, 3′ Untranslated Region.

PFKFB3 plays a critical role in glycolysis in CRC.^11^, ^12^, ^13^, ^14^ The relationship between the endocytosis interaction pathway and regulation of miR-519d-3p was analyzed by DIANA-miRPath data mining.^31^ A total of 25 genes were regulated by miR-519d-3p in this pathway. Cytoscape, a graphical interaction molecule display software, was used to construct a network of interactions between miR-519d-3p and target genes in the Endocytosis interaction pathway. Meanwhile, Bioinformatics TargetScanHuman 8.0 analysis revealed that the PFKFB3–3′-UTR encompassed a binding site for miR-519d-3p (Fig. 4A). The luciferase activity in cells co-transfected with miR-519d-3p mimics and PFKFB3-WT was reduced compared to cells transfected with PFKFB3-MUT, as demonstrated by luciferase assays (Fig. 4B). In addition, miR-519d-3p overexpression or silencing reduced or increased PFKFB3 levels, respectively (Fig. 4C‒E). Furthermore, PFKFB3 silencing could reverse the miR-519d-3p knockdown-mediated PFKFB3 upregulation (Fig. 4F). miR-519d-3p could regulate PFKFB3 and could have a significant effect on CRC.

### Role of PFKFB3 in modulating chemotherapy response and metabolic pathways in crc cells

**Fig. 5:**
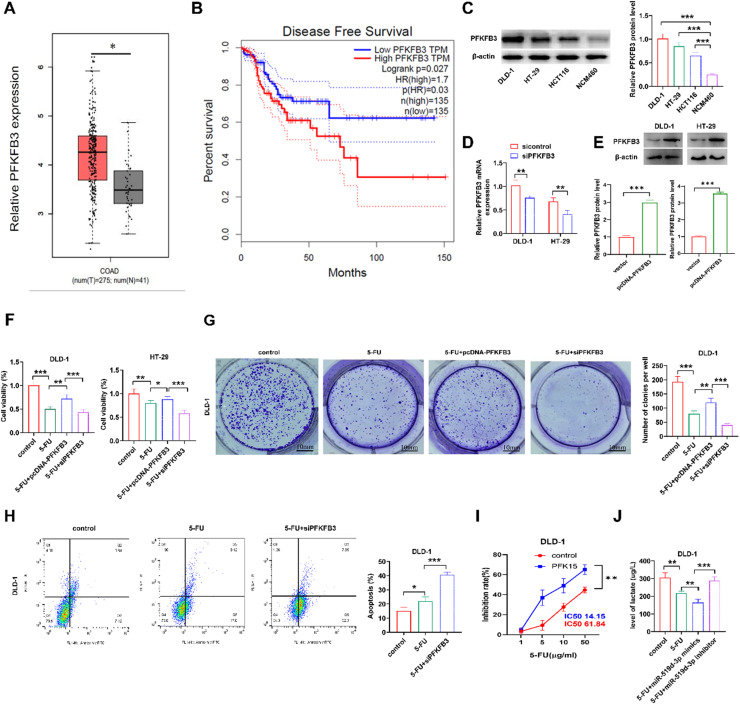
**PFKFB3 attenuates 5-FU chemosensitivity and enhances lactate production in CRC cells.** (A) The differential expression of PFKFB3 between CRC and normal tissues was analyzed by GEPIA database. (B) Bioinformatics analysis in the GEPIA database revealed that high PFKFB3 expression was associated with lesser disease-free survival. (C) The protein expression levels of FPKFB3 were detected using western blot analysis. (D) The mRNA expression levels of PFKFB3 were measured using reverse transcription-quantitative PCR. (E) For the determination of pcDNA-PFKFB3 transfected overexpression protein. (F) Cell viability and (G) colony formation assays were carried out to assess the proliferation ability of PFKFB3-overexpressing or -silencing CRC cells treated with 5-FU. (H) Flow cytometry was used to calculate the apoptosis rate of PFKFB3-depleted DLD-1 cells treated with 5-FU. (I) Cell inhibition rate was determined in CRC cells treated with or without PFK15 (10 μM) and exposed to different concentrations of 5-FU for 24h. (J) Lactate production was determined in miR-519d-3p-overexpressed or -depleted CRC cell lines treated with 5-FU. * *p* < 0.05, ** *p* < 0.01, *** *p* < 0.001. PFKFB3, 6-Phosphofructokinase-2/Frucose-2, 6-Biphosphatase 3; 5-FU, 5-Fluorouracil; CRC, Colorectal Cancer; GEPIA, Gene Expression Profiling Interactive Analysis.

Bioinformatics analysis using the GEPIA database revealed that PFKFB3 was elevated in CRC tissues compared with normal tissues. In addition, the bioinformatics analysis also demonstrated that disease-free survival was significantly decreased (Fig. 5A and B). Furthermore, western blot analysis showed that PFKFB3 was upregulated in CRC cells versus NCM460 cells (Fig. 5C). Subsequently, PFKFB3 was further investigated in vitro for its effect on 5-FU sensitivity in CRC. DLD-1 cells were transfected with pcDNA-PFKFB3 or si-PFKFB3. RT-qPCR analysis showed that DLD-1 cell transfection with si-PFKFB3 showed effectively reduced the PFKFB3 expression (Fig. 5D). and pcDNA-PFKFB3 elevated PFKFB3 expression (Fig. 5E). PFKFB3 overexpression or silencing inhibited or promoted tumor cell proliferation, respectively, following cell treatment with 5-FU (Fig. 5F). These results were further verified by colony formation assays (Fig. 5G), and thus indicating that PFKFB3 knockdown could promote CRC cell apoptosis (Fig. 5H). Additionally, the chemosensitivity of DLD-1 cells to 5-FU treatment at different concentrations was significantly improved in PFKFB3-depleted CRC cells compared with the 5-FU group (Fig. 5I). Furthermore, lactate production was notably reduced in miR-519d-3p-overexpressing DLD-1 cells compared with the 5-FU group. However, lactate secretion was markedly induced in miR-519d-3p-depleted DLD-1 cells (Fig. 5J). Results showed that PFKFB3 attenuated 5-FU sensitivity in CRC cells and enhanced glycolysis.

### The interplay between PFKFB3 and miR-519d-3p in regulating proliferation, apoptosis, and invasion of 5-FU-treated crc cells

**Fig. 6:**
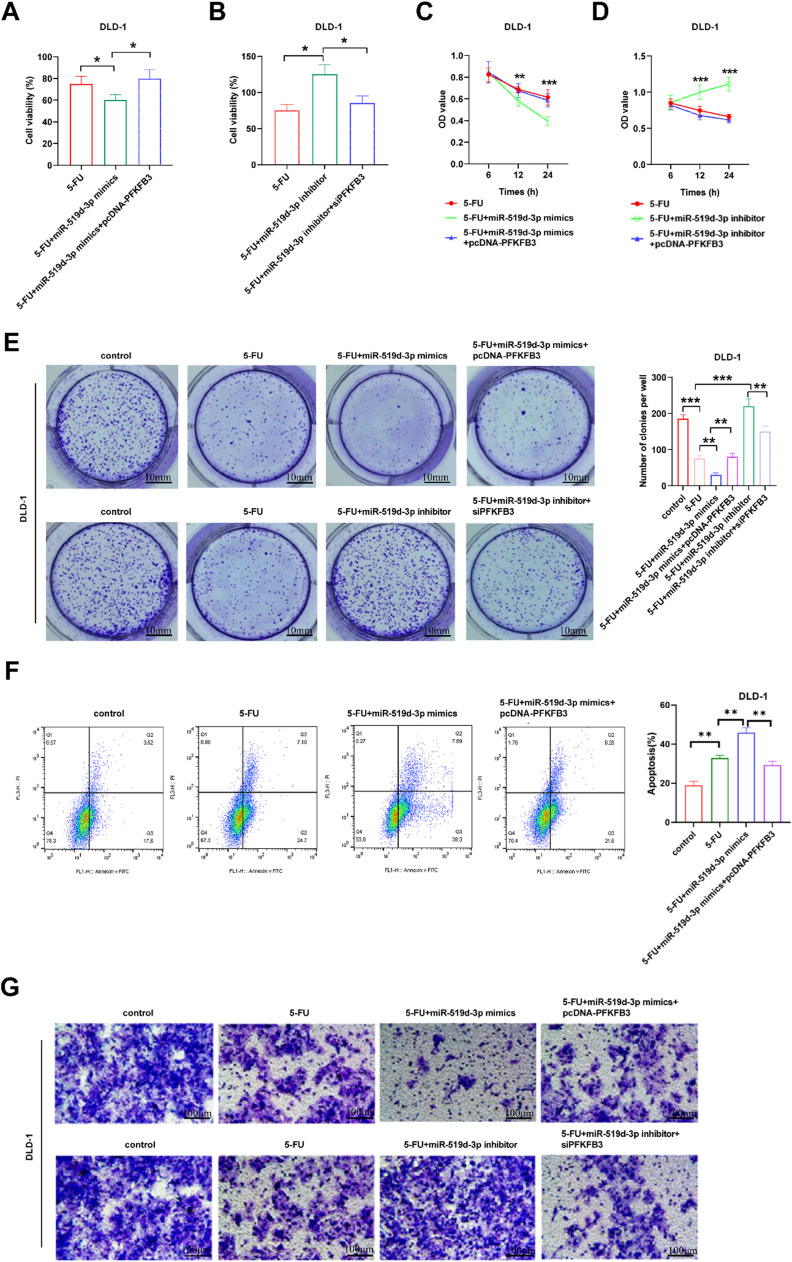
**PFKFB3 inhibits the effect of miR-519d-3p on the chemosensitivity of CRC cells.** (A and B) Cell viability was assessed in CRC cells treated with 5-FU. (C and D) DLD-1 cell viability was significantly higher in the miR-519d-3p mimics + pcDNA-PFKFB3 group compared with the miR-519d-3p mimics group. However, DLD-1 cell viability was reduced in the miR-519d-3p knockdown + PFKFB3 silencing group compared with the miR-519d-3p inhibitor group. (E) 5-FU-treated DLD-1 cell proliferation was assessed using colony formation assays. (F) Flow cytometry was used to determine the apoptosis rate of DLD-1 cells treated with different compounds. (G) The invasion ability (magnification, × 200) of 5-FU-treated DLD-1 cells was enhanced in the PFKFB3 overexpression + miR-519d-3p mimics group compared with the miR-519d-3p mimics group. * *p* < 0.05, ** *p* < 0.01, *** *p* < 0.001. PFKFB3, 6-Phosphofructokin-ase-2/Frucose-2, 6-Biphosphatase 3; miR-519d-3p, miR-519d-3p; CRC, Colorectal Cancer; 5-FU, 5-Fluorouracil.

To validate the effect of miR-519d-3p-targeted regulation of the PFKFB3 axis on CRC cells, functional experiments were carried out. Following cell treatment with 5-FU, the viability and colony formation ability of DLD-1 cells in the miR-519d-3p mimics + pcDNA-PFKFB3 group were enhanced but apoptosis was reduced versus the miR-519d-3p mimics group (Fig. 6A‒F). Furthermore, miR-519d-3p overexpression or silencing significantly attenuated or enhanced, respectively, the invasion ability of 5-FU treated cells, respectively (Fig. 6G). Overall, the aforementioned findings demonstrated that miR-519d-3p could affect CRC cell behavior after 5-FU treatment by directly targeting PFKFB3.

### miR-519d-3p-mediated regulation of 5-FU resistance in vivo

**Fig. 7:**
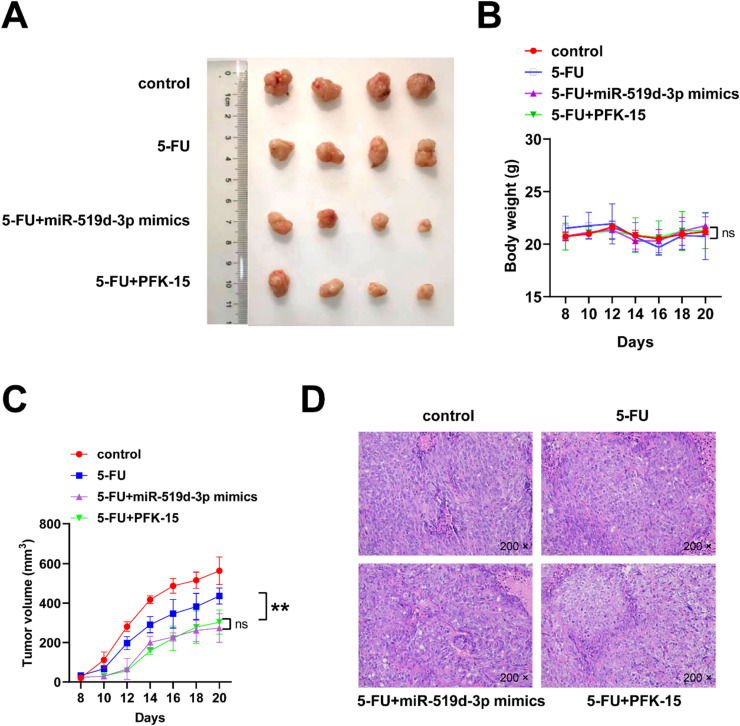
**miR-519d-3p overexpression enhances the chemotherapeutic effect of 5-fluorouracil on colorectal cancer in vivo.** (A) Tumors were excised from mice (*n* = 4) after day 21. (B and C) Body weight and tumor volume were measured every other day. (D) Hematoxylin and eosin staining in xenograft tumor tissues is shown (magnification, × 200). * *p* < 0.05, ** *p* < 0.01. Images were captured at three independent microscopic fields.

By subcutaneously injecting 5 × 10^6^ DLD-1 cells, a mouse model of CRC was created to determine whether miR-519d-3p could increase its chemosensitivity to 5-FU in vivo. Therefore, tumor development was suppressed in the miR-519d-3p mimics + 5-FU group compared with the 5-FU group (Fig. 7A and C). The body weight of mice in the miR-519d-3p mimics + 5-FU group was not decreased versus the other groups (Fig. 7B). Each group was assessed using H&E staining (Fig. 7D).

## Discussion

Chemoresistance is a major factor affecting the treatment efficacy and clinical outcomes of CRC patients,^32^ and studies have shown that about half of stage II or III CRC patients develop resistance or relapse during chemotherapy and that the prognosis of CRC patients is closely related to drug resistance.^33^^,^^34^ Among them, 5-FU has been used for many years and is the first line of chemotherapy, so it is important to search for therapeutic targets to improve 5-FU chemoresistance and provide patients with new therapeutic approaches. The authors first constructed a 5-FU-resistant strain of CRC cells and found that the IC_50_ of the resistant strain was significantly increased by detection, and the growth inhibition of the resistant strain was less restricted as the concentration of 5-FU increased, resulting in a successful resistance model.

The resistance mechanism of 5-FU is complex, ranging from influencing 5-FU metabolizing enzymes, and cellular metabolic process proteins to drug transporter proteins, etc., several studies have shown that miRNA dysregulation and other factors are the current hotspots of 5-FU drug resistance research.^4^^,^^35^^,^^36^ This study focuses on miR-519d-3p, which belongs to the isoform of miR-519d. Ren et al. observed that miR-519 can suppress the proliferation of breast cancer cells by targeting the down-regulation of the human antigen *R*, which has been suggested in previous studies to be closely associated with a variety of malignancies.^37^ Zhou et al. indicated that miR-519d promotes cervical cancer progression and metastasis.^38^ In contrast, studies on the resistance of miR-519d-3p, an isoform of miR-519d, to CRC chemotherapy are lacking. In this study, the authors found that miR-519d-3p expression was downregulated in 5-FU-resistent CRC cells by sequencing as well as RT-qPCR assay. Further functional assays carried out in the present study showed that overexpression of miR-519d-3p increased 5-FU sensitivity, promoted apoptosis, and inhibited cell proliferation (clone formation assay, EDU assay, and Transwell assay); on the contrary, knockdown of miR-519d-3p resulted in decreased 5-FU sensitivity of CRC cells, promoted their proliferation, and decreased apoptosis, indicating further that miR-519d-3p may strengthen the sensitivity of CRC cells to 5-FU. Glucose metabolism is an important energy source process to support tumor cell metabolism, in which glycolysis plays a central role, and PFKFB3 is one of the key enzymes involved in glycolytic metabolism.^39^ In this study, according to the Targetscan website prediction, wherePFKFB3 may be one of the main target genes downstream regulated by miR-519d-3p. By up-regulating and down-regulating miR-519d-3p, determining the mRNA and protein levels of PFKFB3, and constructing a luciferase gene reporter assay, miR-519d-3p was found to negatively phase-regulate the expression of PFKFB3, suggesting that miR-519d-3p can target and regulate PFKFB3. On this basis the authors added PFK-15, an inhibitor of PFKFB3, and the results showed that the negative regulation of PFKFB3 by miR519d-3p could be partially reversed with the use of an inhibitor of PFKFB3, which further demonstrated that miR519d-3p could target and regulate PFKFB3, and the authors then proposed the hypothesis that miR-519d-3p might promote CRC cells' sensitivity to 5-FU by targeting and binding to PFKFB3 and suppressing its expression to promote sensitization of CRC cells to 5-FU. In order to continue to explore the effect of PFKFB3, the target gene of miR-519d-3p on 5-FU sensitivity in CRC cells, the authors performed cellular experiments and found that: 1) The expression of PFKFB3 in CRC cells was higher than that in normal colorectal mucosa; 2) PFKFB3 could enhance the proliferation and reduce the apoptosis of CRC cells under the effect of 5-FU, and thus inhibit the sensitivity of CRC cells to 5-FU.

Previous studies have found that inhibition of miR-330–3p expression promotes glycolytic capacity and promotes CRC resistance to 5-FU.^40^ Deng et al. noted that MiR-488 targeted inhibition of PFKFB3 reduces lactate release and helps to improve the sensitivity of 5-FU and oxaliplatin to CRC cells.^17^ The above studies suggest that the authors the authors can regulate cellular glycolytic metabolism through miRNAs to affect CRC chemosensitivity. Thus, on the basis of the previous experiments, first the authors determined the lactate content in CRC cells under the effect of 5-FU, and found that the lactate content decreased after the intervention, and further decreased after further overexpression of miR-519d-3p. On the basis of the previous experiments, we proceeded to transfect CRC cells with miR-519d-3p mimics to inhibit its downstream target gene PFKFB3, while the transfected miR-519d-3p inhibitor produced the opposite effect, suggesting that miRNA-519d-3p binds PFKFB3 and regulates its expression; subsequently, the authors co-transfected PFKFB3 with miR-519d- 3p cotransfected into CRC cells, and the data showed that the upregulation of PFKFB3 partially reversed the effects of miR-519d-3p on proliferation, apoptosis, and invasion produced in CRC cells under the action of 5-FU. The present findings suggest that microRNA-519d-3p may regulate the biological functions of CRC cells and affect 5-FU sensitivity by targeting the inhibition of PFKFB3.

For the purpose to determine whether miR-519d-3p has similar results in vivo experiments, the authors conducted in-depth nude mouse tumor formation experiments. The authors constructed a nude mouse tumor model, subcutaneously inoculated with CRC cells, and after the tumors grew to a certain extent, they were grouped and treated according to the experimental purpose, and the results showed that the growth of nude mouse tumors was significantly inhibited by the use of 5-FU, and at the same time, the tumor growth was further inhibited by the intra-tumoral injection of miR-519d-3p mimics. This suggests that miR-519d-3p can also inhibit tumor growth in vivo, corroborating with the results of the cell section.

## Conclusion and limitation

Overall, the miR-519d-3p/PFKFB3 axis could be a potential target of a personalized treatment approach for promoting 5-FU sensitivity of CRC. In the future, the authors will add more studies related to 5-FU-resistant cell lines and collect clinical samples at a later stage to continue the research.

## Availability of data and materials

For experiment data acquired please contact corresponding authors.

## Ethics approval

The animal experiment was supported by the Institutional Animal Committee of Nanjing Medical University (Ethical protocol number: 2,203,044 and date: 2022–03–21).

## Patient consent for publication

Not applicable.

## Authors’ contributions

Conceptualization and methodology: Y. Z. and Y.Z.; Software and validation: Y.X. and S.X.; Investigation and resources: J.L.; Data curation: J.L.; Writing-original draft preparation: Y.Z. and L.C.; Writing-review and editing: J.D.; Visualization: D.W.; Supervision: G.X. and L.W.; Funding acquisition: K.W., J.L, and L.C. All authors have read and agreed to the published version of the manuscript.

## Funding

This work was supported by the “Six Ones Project” Top-notch Talent Project for High-level Health Talents of Jiangsu Province to JP (LGY2019078); the 789 10.13039/100022958Outstanding Talent Program of SAHNMU to JD (789ZYRC202090147); and the Nanjing Medical University Science and Technology Development Fund (NMUB20220002).

## Declaration of competing interest

The authors declare no conflicts of interest.

## Appendix Supplementary materials

Image, application 1

## References

[1] Sung H., Ferlay J., Siegel R.L., Laversanne M., Soerjomataram I., Jemal A. (2021). Global Cancer statistics 2020: GLOBOCAN estimates of incidence and mortality worldwide for 36 cancers in 185 countries. CA Cancer J Clin.

[2] Catalano T., D'Amico E., Moscatello C., Di Marcantonio M.C., Ferrone A., Bologna G. (2021). Oxidative distress induces wnt/β- catenin pathway modulation in colorectal cancer cells: perspectives on APC retained functions. Cancers (Basel).

[3] Dong S., Liang S., Cheng Z., Zhang X., Luo L., Li L. (2022). ROS/PI3K/akt and wnt/beta-catenin signalings activate HIF-1alpha-induced metabolic reprogramming to impart 5-fluorouracil resistance in colorectal cancer. J Exp Clin Cancer Res.

[4] Blondy S., David V., Verdier M., Mathonnet M., Perraud A., Christou N. (2020). 5-Fluorouracil resistance mechanisms in colorectal ancer: from classical pathways to promising processes. Cancer Sci.

[5] Yang I.-P., Yip K.-L., Chang Y.-T., Chen Y.-C., Huang C.-W., Tsai H.-L. (2023). MicroRNAs as predictive biomarkers in patients with colorectal cancer receiving chemotherapy or chemoradiotherapy: a narrative literature review. Cancers (Basel).

[6] He B., Zhao Z., Cai Q., Zhang Y., Zhang P., Shi S. (2020). miRNA-based biomarkers, therapies, and resistance inCancer. Int J Biol Sci.

[7] Liang J., Liu Y., Zhang L., Tan J., Li E., Li F. (2019). Overexpression of microRNA-519d-3p suppressed he growth of pancreatic cancer cells by inhibiting ribosomal protein S15A-mediated wnt/β-catenin signaling. Chem Biol Interact.

[8] Li Y.-Y., Shao J.-P., Zhang S.-P., Xing G.-Q., Liu H.-J. (2018). miR-519d-3p inhibits cell proliferation and invasion of gastric cancer by downregulating B-cell lymphoma 6. Cytogenet Genome Res.

[9] Jiang L., Shi S., Li F., Shi Q., Zhong T., Zhang H. (2021). miR-519d-3p/HIF 2α axis increases the chemosensitivity of human cervical cancer cells to cisplatin via inactivation of PI3K/AKT signaling. Mol Med Rep.

[10] Chen R., Zhou S., Chen J., Lin S., Ye F., Jiang P. (2020). LncRNA BLACAT1/miR-519d-3p/CREB1 axis mediates proliferation, apoptosis, migration, invasion, and drug-resistance n colorectal cancer progression. Cancer Manag Res.

[11] Da Q., Huang L., Huang C., Chen Z., Jiang Z., Huang F. (2023). Glycolytic regulatory enzyme PFKFB3 as a prognostic and tumor microenvironment biomarker in human cancers. Aging (Albany NY).

[12] Sun J., Zhang P., Yin T., Zhang F., Wang W. (2020). Upregulation of LncRNA PVT1 facilitates pancreatic ductal adenocarcinoma cell progression and glycolysis by regulating MiR-519d-3p and HIF-1A. J Cancer.

[13] Sun L., Li J., Yan W., Yao Z., Wang R., Zhou X. (2021). H19 promotes aerobic glycolysis, proliferation, and immune escape of gastric cancer cells through the microRNA-519d-3p/lactate dehydrogenase A axis. Cancer Sci.

[14] Peng F., Li Q., Sun J.-Y., Luo Y., Chen M., Bao Y. (2018). PFKFB3 is involved in breast cancer proliferation, migration, invasion and angiogenesis. Int J Oncol.

[15] Minchenko O.H., Tsuchihara K., Minchenko D.O., Bikfalvi A., Esumi H. (2014). Mechanisms of regulation of PFKFB expression in pancreatic and gastric cancer cells. World J Gastroenterol.

[16] Long Q., Zou X., Song Y., Duan Z., Liu L. (2019). PFKFB3/HIF-1α feedback loop modulates sorafenib resistance in hepatocellular carcinoma cells. Biochem Biophys Res Commun.

[17] Deng X., Li D., Ke X., Wang Q., Yan S., Xue Y. (2021). Mir-488 alleviates chemoresistance and glycolysis of colorectal cancer by targeting PFKFB3. J Clin Lab Anal.

[18] Sharda N., Ikuse T., Hill E., Garcia S., Czinn S.J., Bafford A. (2021). Impact of andrographolide and melatonin combinatorial drug therapy on metastatic colon cancer cells and organoids. Clin Med Insights Oncol.

[19] Wang M., Yu K., Fu W., Yang L. (2024). The combination of SHP099 inhibits the malignant biological behavior of l-OHP/5-FU-resistant colorectal cancer cells by regulating energy metabolism reprogramming. Biochem Biophys Res Commun.

[20] Wang B., Lu F.-Y., Shi R.-H., Feng Y.-D., Zhao X.-D., Lu Z.-P. (2018). MiR-26b regulates 5-FU-resistance in human colorectal cancer via down-regulation of pgp. Am J Cancer Res.

[21] Zhang G., Luo X., Wang Z., Xu J., Zhang W., Chen E. (2022). TIMP-2 regulates 5-fu resistance via the ERK/MAPK signaling pathway in colorectal cancer. Aging (Albany NY).

[22] Chen Z., Huang M., You J., Lin Y., Huang Q., He C. (2022). Circular RNA hsa_circ_0023404 promotes the proliferation, migration and invasion in endometrial cancer cells through regulating miR-217/MAPK1 axis. Eur J Med Res.

[23] Zhang W., Zhu Y., Zhou Y., Wang J., Jiang P., Xue L. (2022). miRNA-31 increases radiosensitivity through targeting STK40 in colorectal cancer cells. Asia Pac J Clin Oncol.

[24] Song Y., Li L., Ou Y., Gao Z., Li E., Li X. (2014). Identification of genomic alterations in oesophageal squamous cell cancer. Nature.

[25] Yang Y., Yuan H., Zhao L., Guo S., Hu S., Tian M. (2022). Targeting the miR-34a/LRPPRC/MDR1 axis collapse the chemoresistance in P53 inactive colorectal cancer. Cell Death Differ.

[26] Hong J.W., Kim J.M., Kim J.E., Cho H., Kim D., Kim W. (2020). MiR-4435 is an UQCRB-related circulating miRNA in human colorectal cancer. Sci Rep.

[27] Chen Y., Guo Y., Yuan M., Guo S., Cui S., Chen D. (2023). USP4 promotes the proliferation and glucose metabolism of gastric cancer cells by upregulating PKM2. PLoS One.

[28] Zhou F., Gao H., Shang L., Li J., Zhang M., Wang S. (2023). Oridonin promotes endoplasmic reticulum stress via TP53-repressed TCF4 transactivation in colorectal cancer. J Exp Clin Cancer Res.

[29] Guo L., Cai Y., Wang B., Zhang F., Zhao H., Liu L. (2023). Characterization of the circulating transcriptome expression profile and identification of novel miRNA biomarkers in hypertrophic cardiomyopathy. Eur J Med Res.

[30] Gómez-Acebo I., Llorca J., Alonso-Molero J., Díaz-Martínez M., Pérez-Gómez B., Amiano P. (2023). Circulating miRNAs signature on breast cancer: the MCC-Spain project. Eur J Med Res.

[31] Cheng J., Zhuo H., Wang L., Zheng W., Chen X., Hou J. (2020). Identification of the combinatorial effect of miRNA Family regulatory network in different growth patterns of GC. Mol Ther Oncolytics.

[32] Longley D.B., Johnston P.G. (2005). Molecular mechanisms of drug resistance. J Pathol.

[33] Huang X., Ke K., Jin W., Zhu Q., Zhu Q., Mei R. (2022). Identification of genes related to 5-fluorouracil based chemotherapy for colorectal cancer. Front Immunol.

[34] Malier M., Gharzeddine K., Laverriere M.-H., Marsili S., Thomas F., Decaens T. (2021). Hypoxia drives dihydropyrimidine dehydrogenase expression in macrophages and confers chemoresistance in colorectal cancer. Cancer Res. 2021.

[35] Vodenkova S., Buchler T., Cervena K., Veskrnova V., Vodicka P., Vymetalkova V. (2020). 5-fluorouracil and other fluoropyrim-idines in colorectal cancer: past, present and future. Pharmacol Ther.

[36] Escalante P.I., Quiñones L.A., Contreras H.R (2021). Epithelial-mesenchymal transition and MicroRNAs in colorectal cancer chemoresistance to FOLFOX. Pharmaceutics.

[37] Ren L., Li Y., Zhao Q., Fan L., Tan B., Zang A. (2020). miR-519 regulates the proliferation of breast cancer cells via targeting human antigen R. Oncol Lett.

[38] Zhou J.Y., Zheng S.R., Liu J., Shi R., Yu H.-L., Wei M. (2016). MiR-519d facilitates the progression and metastasis of cervical cancer through direct targeting Smad7. Cancer Cell Int.

[39] Kotowski K., Rosik J., Machaj F., Supplitt S., Wiczew D., Jabłońska K. (2021). Role of PFKFB3 and PFKFB4 in cancer: genetic basis, impact on disease development/progression, and potential as therapeutic targets. Cancers (Basel).

[40] Gao S.-J., Ren S.-N., Liu Y.-T., Yan H.-W., Chen X.-B. (2021). Targeting EGFR sensitizes 5-Fu-resistant colon cancer cells through modification of the lncRNA-FGD5-AS1-miR-330-3p-hexokinase 2 axis. Mol Ther Oncolytics.

