# Supplementary material for: Investigating the role of MicroRNA-519d-3p in enhancing chemosensitivity of colorectal cancer cells to 5-Fluorouracil through PFKFB3 targeting
Source: Clinics (Sao Paulo). 2025 Feb 26;80:100606. doi: 10.1016/j.clinsp.2025.100606 (PMC11910362; doi:10.1016/j.clinsp.2025.100606)
Supplement: Supplementary file 1 [file mmc1.pdf]

EDITORIAL CERTIFICATE

美辑编译
www.mjeditor.com

NO.MJ202403260715

To whom it may concern:Yangyang Zhang, Yiqing Zhang, Yanan Xiao, Shufen Xu, Jie Li, Juan Li, Lisha Chang, Jie Ding, Di Wu, Li Wang, Guangxu Xu, Keming Wang

The paper“Investigating the Role of MicroRNA-519d-3p in Enhancing Chemosensitivity of Colorectal Cancer Cells to 5-Fluorouracil through PFKFB3 Targeting”

was edited by MJ Language Editing Services.

Sincerely,

Biji MJ Webshop Support

MEIJI LANGUAGE EDITING
4403030526501
